# Metabolomics of cereals under biotic stress: current knowledge and techniques

**DOI:** 10.3389/fpls.2013.00082

**Published:** 2013-04-23

**Authors:** Dirk Balmer, Victor Flors, Gaetan Glauser, Brigitte Mauch-Mani

**Affiliations:** ^1^Institute of Biology, University of NeuchâtelNeuchâtel, Switzerland; ^2^Metabolic Integration and Cell Signaling Group, Plant Physiology Section, Departamento de Ciencias Agrarias y del Medio Natural, Universitat Jaume ICastellón, Spain; ^3^Chemical Analytical Service of the Swiss Plant Science Web, University of NeuchâtelNeuchâtel, Switzerland

**Keywords:** chemical analytical techniques, chemical defense, metabolic profile, monocots, phytoalexins, secondary metabolites

## Abstract

Prone to attacks by pathogens and pests, plants employ intricate chemical defense mechanisms consisting of metabolic adaptations. However, many plant attackers are manipulating the host metabolism to counteract defense responses and to induce favorable nutritional conditions. Advances in analytical chemistry have allowed the generation of extensive metabolic profiles during plant-pathogen and pest interactions. Thereby, metabolic processes were found to be highly specific for given tissues, species, and plant-pathogen/pest interactions. The clusters of identified compounds not only serve as base in the quest of novel defense compounds, but also as markers for the characterization of the plants' defensive state. The latter is especially useful in agronomic applications where meaningful markers are essential for crop protection. Cereals such as maize make use of their metabolic arsenal during both local and systemic defense responses, and the chemical response is highly adapted to specific attackers. Here, we summarize highlights and recent findings of metabolic patterns of cereals under pathogen and pest attack.

## INTRODUCTION

The major part of the world’s food supply depends on the production of cereal crops such as rice, maize, wheat, barley, sorghum, oat, and millet. These crops are constantly jeopardized by biotic stressors such as viruses, bacteria, fungi, or herbivores leading to severe yield losses and therefore to eminent economic problems. For instance, the hemibiotrophic fungus *Colletotrichum graminicola*, the causal agent of maize anthracnose, is responsible for annual losses of up to one billion dollars in the U.S. (Frey et al., 2011). Hence, understanding the defense mechanisms of cereals is crucial in developing sustainable crop enhancement programs. Intriguingly, despite the large variety of potential attackers, only few pathogens and pests are able to successfully parasitize a particular plant species. This corroborates the fact that plants employ a highly intricate defense system that is capable of fending off the majority of attackers. Plant immunity is multilayered and consists of pre-formed, constitutive as well as inducible defense mechanisms (Pieterse et al., 2009). Besides physical pre-formed barriers such as the cell wall, plants also possess highly effective pre-formed chemical defenses called phytoanticipins (González-Lamothe et al., 2009). Those are constitutively present products of secondary plant metabolism. They represent a first defense layer and are released and activated as antimicrobial compounds upon pathogen entry. A diverse family of phytoanticipins is composed of the so-called saponins, secondary metabolites that can be found in many plant species but particularly in dicots. Intriguingly, with the exception of oats, cereals are generally deficient in saponins (Osbourn, 2003). In addition to pre-formed chemical defenses, plants also employ antimicrobial compounds that are induced only upon pathogen or pest attack. These compounds are defined as phytoalexins (Hammerschmidt, 1999), antimicrobial compounds whose induction is mediated by a pathogen-triggered activation of enzymes involved in their synthesis. Usually, phytoalexins possess rather unspecific inhibitory effects on a wide range of different pathogens.

**FIGURE 1:**
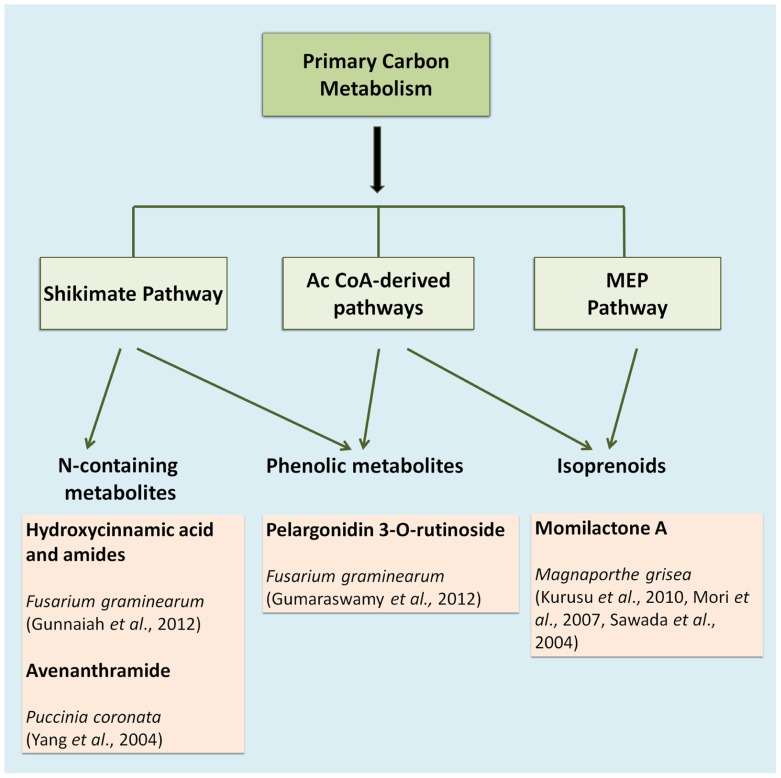
**Main metabolic pathways involved in cereal defense**.

The compounds that constitute the chemical defense arsenal of plants stem from various metabolic pathways, and can be roughly categorized in three major groups, namely alkaloids (e.g., the indole alkaloid camalexin), isoprenoids (e.g., diterpenes), and shikimates (e.g., flavonoids; Großkinsky et al., 2012; **Figure 1**). Alkaloids are mainly synthesized via the citric acid cycle or shikimate pathway; isoprenoids are synthesized via the acetate-mevalonate or methylerythritol phosphate pathway, whereas phenylpropanoids are mainly built over the shikimate pathway (Großkinsky et al., 2012). The entire set of metabolites synthesized via these and various other pathways is defined as the plant’s metabolome, which may be viewed as the biochemical phenotype of a given plant tissue. In metabolomic analysis, such biochemical phenotypes can be qualitatively and quantitatively profiled on a large scale. In recent years, metabolite profiling has become a standard research tool for high-throughput diagnostics in various plant science applications, such as phenotyping of different species and analysis of resistance traits or responses to herbicides (Schauer and Fernie, 2006). In concert with transcriptomics, metabolomics has become an indispensable tool in screening crop germplasm collections during crop breeding programs (Langridge and Fleury, 2011). A plant’s metabolome plays an important role in a wide range of physiological processes, and current research on plant stress responses greatly benefits from recent advances in metabolite profiling methods (Großkinsky et al., 2012).

Apart from some recent articles (Allwood et al., 2010; Du Fall and Solomon, 2011) very few metabolomic studies on the interactions between biotic stressors and plants, especially cereals, have been reported. In this review, the role of metabolites in response to pathogens is elucidated, along with their role in herbivore defense. Moreover, recent advances in metabolite profiling and analysis techniques are summarized, giving a comprehensive overview of the current methods available for metabolomic analysis in cereals.

## METABOLOMIC RESPONSES OF CEREALS TO NECROTROPHIC PATHOGENS

A model for a metabolomic study applied to fungal diseases must take into account several criteria such as: an accurate identification of the compounds or at least a putative identification of metabolites; a statistical significance within the studied variations; a strong change in concentration between resistant/susceptible plant-pathogen metabolome; and finally, if possible, assignation to a known plant defense pathway.

As an example, *Fusarium* head blight (FHB) is one of the most devastating diseases that affect several monocotyledonous plants such as barley, maize, wheat, and triticale (Choo, 2006). *Fusarium* is a necrotrophic pathogen and uses mycotoxins to kill the plant tissue before being able to feed on the host cells. Resistance to FHB is associated with more than 100 quantitative trait loci (QTLs) distributed along the seven chromosomes of barley and wheat. However, only the function of the *Qfhs.ndsu-3BS* QTL has been clearly defined in resistance, since it is involved in the detoxification of the mycotoxin deoxynivalenol (DON) into its less toxic glucoside, DON-3-O-glucoside (Lemmens et al., 2005). In such a case, the study of resistance controlled by polygenes with low heritability that changes depending on environment, location and year, is time consuming and not very efficient. Fortunately, the existence of metabolomic resources is a very valuable tool to search for metabolites with resistance-related potential in such complicated landscapes. Using liquid chromatography coupled to mass spectrometry (LC-MS) Bollina et al. (2010) identified 496 metabolites in barley that were overrepresented in a metabolomic analysis of a resistant cultivar compared to a susceptible one. They assigned a putative identity based on the accurate mass, fragmentation pattern and the number of carbons in the formula to these metabolites. Interestingly, most of the metabolites from the resistance cluster (RR) were derived from the phenylpropanoid, flavonoid, fatty acid, and terpenoid metabolic pathways (**Figure 1**). Their putative role in resistance was further confirmed by *in vitro* bioassays for antifungal activity. Among the RR cluster several precursors of kaempferol were identified to play a relevant role in the enhanced defense capacity of the resistant cultivar (Bollina et al., 2010).

To study the role of metabolites participating in resistance identical genetic backgrounds should be used, since differences in the metabolites may derive from differences in the plant genotypes. Furthermore, it is also possible to find pathogen-derived metabolites. However, it is expected that resistance is also associated to lower levels of fungal growth and therefore, the selection criteria based on the higher abundance of metabolites in the resistant genotypes makes the selection of fungal compounds as resistance metabolites rather unlikely.

**FIGURE 2:**
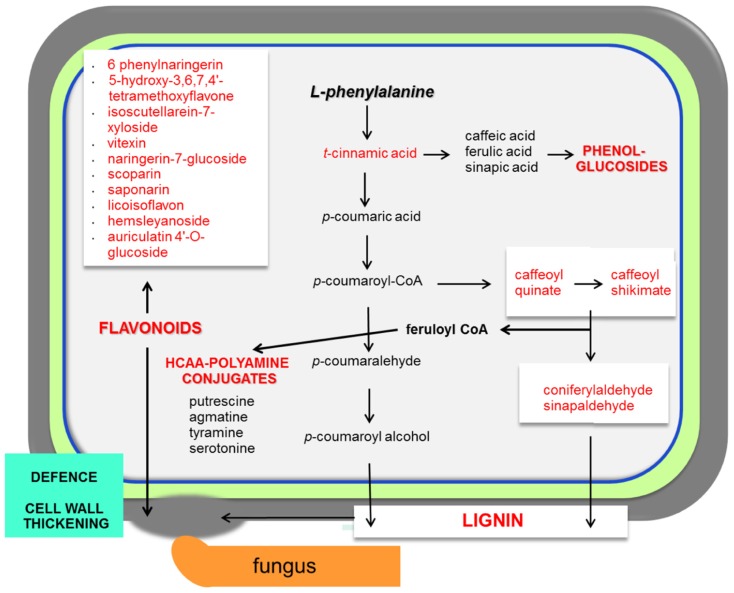
**Necrotrophs interacting with barley and wheat**. The main responses of cereals during necrotrophic interactionsare focused in the activation of the phenylpropanoid pathway. The infected plant accumulates lignin, phenol-glucosides, hydroxycinnamic acid conjugated with polyamine derivatives (HCAA; Gunnaiah et al., 2012) and also flavonoids. Abundant metabolites in cereal–necrotoph interactions are represented in red. Pathways that are activated during the interaction with necrotrophs are represented in bold red. This model is based on interactions between barley/wheat and *Fusarium* sp. (Bollina et al., 2010)

The range of resistance of barley spikelets to *F. graminearum* is classified as a type II resistance (Schroeder and Christensen, 1963). The *Fusarium* mutant trichothecene-non-producing (*tri5*-) fails to spread within inoculated spikes in wheat (Jansen et al., 2005). The combined system of resistant and susceptible barley, together with trichothecene-producing and non-producing *F. graminearum* strains, is a good model to study the metabolic responses that regulate resistance in barley to this fungal disease (Kumaraswamy et al., 2012). This research revealed the existence of constitutive resistance-related (RRC) and induced resistance-related (RRI) metabolites. Examples of specific RRC compounds with elevated levels found in resistant barley are coniferylaldehyde, pelargonidin 3-O-rutinoside, vitexin, and 8E-heptadecenoic acid (**Figure 2**; Kumaraswamy et al., 2012). Even more relevant was the finding that indole acetic acid, picolinic acid, and a glucoside of feruloyl alcohol showed higher concentrations in response to the trichothecene producing strain in the resistant barley.

In wheat, the Fhb1 (*Fusarium* head blight 1 resistance locus) QTL is believed to be responsible for resistance to the spread of *F. graminearum* within the spikes. This resistance is mainly attributed to the activation of the phenylpropanoid, terpenoid, and fatty acid metabolic pathways (**Figure 1**) in addition to the detoxification of DON to DON-3G. Non-targeted proteomics based on 2D gel electrophoresis combined with LC-MS/MS have been applied to this plant-pathogen system (Gunnaiah et al., 2012). Proteomic studies confirmed the implication of these pathways but also the relevant role of the oxidative burst and the accumulation of PR-1, 1,3-β-glucanases, chitinases, and PR-10 proteins. In addition, methionine synthase, S-adenosylmethionine synthase, 5,10-methylene-tetrahydrofolate reductase, and adenosylhomocysteine hydrolase, that increase the activity of the ethylene and phenylpropanoid pathway, were shown to be more active in the resistant lines of wheat. However, the participation of *Fhb1* in resistance is mainly due to its involvement in the regulation of the phenylpropanoid pathway. This is a good example demonstrating that proteo-metabolomic studies are not only restricted to the genetics of a given QTL (Gunnaiah et al., 2012). These studies also revealed that jasmonic acid isoleucine (JA-Ile and JA together with HCAAs (hydroxycinnamic acid amide, conjugates of phenol-polyamines) such as coumaroyl putrescine/agmatine and feruloyl putrescine/agmatine overaccumulate in resistant wheat cultivars (**Figure 2**; Gunnaiah et al., 2012).

Another model cereal studied in connection with interactions between plants and necrotrophic fungi is maize. Recently, a new function for benzoxazinones (BX) in the resistance against the necrotrophic fungus *Setosphaeria turcica* was elucidated (Ahmad et al., 2011). An accepted mode of action is attributed to the toxicity of the aglucones when the BX-glucosides are hydrolyzed by plastid-targeted β-glucosidases (Morant et al., 2008). The use of ultra-high pressure LC (UHPLC) coupled to QTOFMS is a valuable tool to determine the occurrence of these compounds under various experimental conditions (Ahmad et al., 2011; Glauser et al., 2011).

## METABOLOMIC RESPONSES OF CEREALS TO BIOTROPHIC PATHOGENS

*Magnaporthe oryzae* shows a hemibiotrophic life style characterized by apparently unaffected host cells that retain the ability to plasmolyse (Koga et al., 2004). In contrast, during incompatible interactions of rice cells with the fungus, the cells lose membrane integrity and the ability to plasmolyse, showing granulation and other symptoms usually associated to a necrotrophic mechanism of infection. Therefore, the degree of incompatibility conditions the lifestyle of *Magnaporthe oryzae*, which behaves only as a pure biotroph in fully compatible interactions. *Magnaporthe oryzae* infects plant cells via germinating conidia at the leaf surface. The germtube produces an appressorium from which a penetration peg grows into the cell. The penetration peg gives rise to numerous invasive biotrophic hyphae that are separated from the host cytoplasm by a plant-generated membrane. Fungal progression to neighboring cells is likely taking place through plasmodesmata since plant cell wall integrity is not disturbed (Kankanala et al., 2007). In addition, the biotrophic strategy of rice blast is different when it invades the first layer of cells or subsequent cells. The metabolic interplay during such a finely controlled process is difficult to study.

**FIGURE 3:**
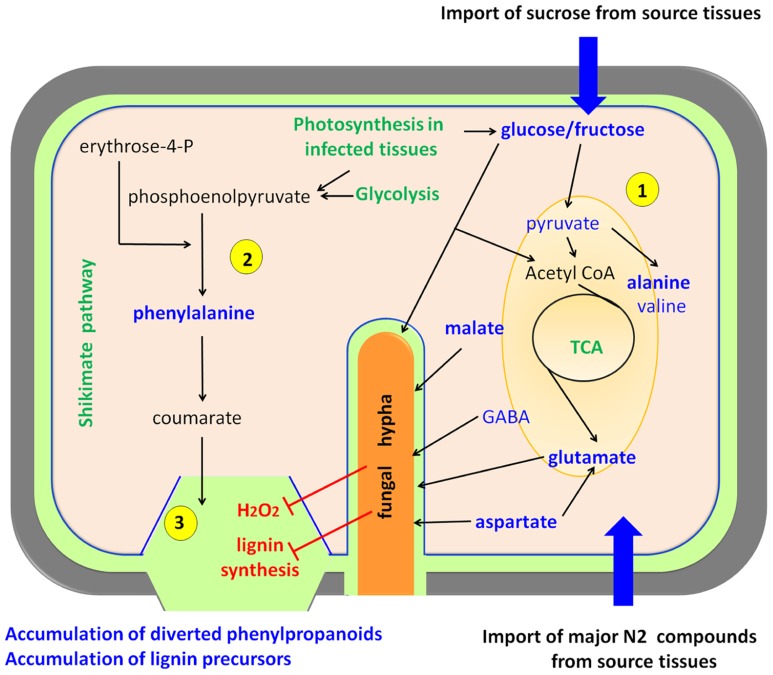
**Biotrophs interacting with rice**. Biotrophic pathogens feed from living cells forcing the host to increase its primary metabolism. Plant cells over-compensate the carbon and nitrogen depletion by acting as a sink for organic compounds that are imported from plant source tissues, and also by increasing their photosynthesis, gluconeogenesis, and glycolysis **(1)** The main pathways activated upon fungal infection are the tricarboxylic acid cycle (TCA) and the glycolysis. On the other hand, plant defense attempts to stimulate the shikimate pathway and lignin biosynthesis **(2)**, but the fungus hijacks this processes with the help of effectors (Mentlak et al., 2012) and by inhibiting oxidative crosslinking of phenolics, thus leading to an over-accumulation of free phenylpropanoids and lignin precursors **(3)** (Parker et al., 2009). Upregulated metabolic pathways are depicted in green, compounds present in high abundance upon infection in blue, and processes inhibited by the pathogen in red. This model is essentially based on rice-*Magnaporthe grisea* interactions.

In a detailed study of the metabolic interplay between rice and *Magnaporthe grisea*, two major findings that define the metabolic reprogramming were observed (**Figure 3**). Infected leaf tissues displaying lesions accumulated Ala (alanine), Pro (proline), His (histidine), Cys (cysteine), and Trp (tryptophan) among other amino acids, and sucrose, malate, fructose, and glucose (Parker et al., 2009; Jones et al., 2011). This has been observed in susceptible rice genotypes suggesting that infected leaves with visible lesions become metabolic photosynthetic sinks (Parker et al., 2009). This observation fits well with the biotrophic lifestyle of *Magnaporthe grisea*; however, there is also an accumulation of phenylpropanoid and phenolic compounds that resembles the plant-necrotroph responses described above. A very likely explanation is that rice is triggering cell wall reinforcements that are less pronounced in susceptible phenotypes due to the reduced generation of H_2_O_2_ (**Figure 3**). This causes a deficit in phenolic cross-linking in the cell compared to resistant phenotypes. Finally during the latter stages of infection, leakage of nutrients from dying cells might act as energy supply for the sporulation process of the fungus (Parker et al., 2009).

Again, the combination of compatible and incompatible strains of *Magnaporthe grisea* provides a perfect scenery to study metabolic reprogramming related to defense in rice. Jones et al. (2011) used MS and nuclear magnetic resonance (NMR)-based metabolomics to assess the response to the fungus at different time-points after infection. Among many other interesting compounds, they found that the major changes in each interaction involved malate, glutamine, Ala, Pro, cinnamate, and sugars. Interestingly, they proposed that fungus-triggered high levels of Ala may be responsible for cell death to facilitate *Magnaporthe grisea* invasion. These observations suggest that the negation of such responses may cause incompatibility in the interaction, thereby stopping the infection. Despite such attractive conclusions further studies are needed for a final demonstration of the roles of Ala in the establishment of compatibility (Jones et al., 2011).

## METABOLOMICS IN DEFENSE AGAINST HERBIVORES AND NEMATODES

Plants also produce specific secondary metabolites to protect themselves against potential pest herbivores or nematodes. The importance of such metabolites is reflected in the extensive portion of the genome allocated to genes involved in primary or secondary metabolism, which has been estimated to be 25% of the rice (*Oryza sativa* L. ssp. *japonica*) genome as an example (Goff et al., 2002).

Metabolomics studies in the classical sense are scarce in cereals and even more so in the field of cereal–herbivore interactions. Although numerous QTLs linked to insect herbivore resistance have been identified, the genetic basis responsible for these traits is in most cases unknown. Most cereal metabolites with insecticidal and/or nematicidal properties have also been shown to inhibit the growth of pathogens and are derived from the same chemical classes as the ones active against microbes.

### BENZOXAZINOIDS

The best-investigated anti-herbivore secondary metabolites in cereals are the benzoxazinoids, molecules with a 2-hydroxy-2*H*-1,4-benzoxazin-3(4*H*)-one skeleton. Among them, the hydroxamic acids are the most active ones (Niemeyer, 2009). In the plants, these molecules are usually glucosylated and their activity rises after enzymatic hydrolyses to an aglucone. The biosynthetic pathway leading to their generation is well known (Niemeyer, 1988; Sicker and Schulz, 2002).

Erb et al. (2009) investigated the reaction of maize (*Zea mays*) to belowground attack by the western corn rootworm *Diabrotica virgifera virgifera *on the defensive capacity of the aboveground organs against another herbivore insect pest, *Spodoptera littoralis *and also monitored the accumulation of defensive metabolites following challenge of the leaves with *Spodoptera*. Quantification of metabolites in the leaves by HPLC-DAD (high-performance LC with diode-array detection) revealed a direct induction of 2,4-dihydroxy-7-methoxy-1,4-benzoxazin-3-one (DIMBOA) following root attack and an additional increase upon challenge with *Spodoptera*. The various treatments did not affect the levels of DIMBOA-glucoside (DIMBOA-Glc). Analysis of phenolic compounds by ultra performance LC (UPLC)-MS showed that caffeic acid production was suppressed following infestation by either of the insects. However, chlorogenic acid was induced directly only by *Spodoptera*, but prior infestation of the root system with *Diabrotica* primed the leaf tissues to produce more chlorogenic acid upon *Spodoptera* challenge. Interestingly, direct induction of DIMBOA and priming of chlorogenic acid accumulation in the leaves can be mimicked by applying abscisic acid (ABA) to the roots of the maize plants. However, the involvement of additional metabolites or mechanisms must be assumed since root treatment of maize plants with ABA alone did not induce resistance against against *Spodoptera littoralis *(Erb et al., 2009). Recent findings uncovered a dual role of BX in inducible herbivore resistance (Glauser et al., 2011). Both *Spodoptera littoralis* and *Spodoptera frugiperda* were shown to be able to detoxify DIMBOA, which was rapidly released from its corresponding glucoside in the primary response against herbivores. In contrast, the highly unstable 2-hydroxy-4,7-dimethoxy-1,4-benzoxazin-3-one (HDMBOA), which is released in a second step during herbivore attack, functions as deterrent to *Spodoptera littoralis* and *Spodoptera frugiperda* and is quickly degraded in the insect guts.

Besides their direct toxic effect, BX seem to also have a regulatory role in innate immunity. Ahmad et al. (2011) compared the expression of basal resistance in BENZOXAZINELESS1 (*BX1*) wild type and *bx1* mutant maize lines. The *bx1* mutants, besides being less resistant to the fungal pathogen *Setosphaeria turcica*, supported a better development of the cereal aphid *Rhopalosiphum padi. *Already during early infestation stages by *R. padi *an increased accumulation of DIMBOA-Glc, DIMBOA itself, and HDMBOA-glucoside (HDMBOA-Glc) was measured in the leaves. Leaf infiltration with chitosan, an elicitor of defense produced by deacetylation of chitin, a structural element in the skeleton of insects or the cell wall of fungi, also enhanced the accumulation of DIMBOA and HDMBOA-Glc. The expression of genes in the biosynthetic pathway leading to BX however was downregulated downstream of BX1 by chitosan. Additionally, in *bx1* mutants, callose deposition elicited by chitosan infiltration was reduced compared to wild type. These findings all point toward a role for DIMBOA as a signal in the regulation of maize innate immunity.

An additional role for BX in cereal defense has been suggested in the protection against nematodes. Rye (*Secale cereale*) planted as an annual winter cover crop, is able to reduce insect and nematode infestation in the following crop (Zasada et al., 2005). Since a biocidal action of low molecular weight aliphatic organic acids from such rye plants against *Meloidogyne incognita* had been ruled out (McBride et al., 2000), other possibly involved metabolites were tested. Based on reports that the BX DIBOA (2,4-dihydroxy-1,4-benzoxazin-3-one) and its breakdown product benzoxazolin-2(3*H*)-one (BOA) as well as DIMBOA and its degradation product 6-methoxy-BOA (MBOA) in rye had allelopathic properties (Barnes and Putnam, 1987; Rice et al., 2005), these substances were also tested as to their influence on nematodes (Zasada et al., 2005). DIBOA was shown to be more toxic than DIMBOA. In contrast to corn and wheat, where DIMBOA is the main metabolite, in rye, DIBOA predominates (Friebe, 2001; Rice et al., 2005), making it a possible candidate for nematode control. DIBOA caused a higher mortality than DIMBOA in both plant parasitic nematodes *Xiphinema americanum* and *Meloidogyne incognita, *respectively, whereas eggs were less affected than adults and juveniles (Zasada et al., 2005). Such *in vitro* toxicity studies have to be relativized since it was shown later by the same research group that, based on the fate of DIBOA in agricultural soils, the actually present concentration might be too low to be a major factor in containing nematode populations.

### FLAVONOIDS

Flavonoids such as the C-glycosyl flavones maysin and apimaysin found in corn silk for instance have been shown to inhibit the growth of corn earworm (*Helicoverpa zea)* larvae (Lee et al., 1998). Based on QTL analysis, 55– 65% of phenotypic variance against the corn earworm could be attributed to maysin or apimaysin, respectively. Interestingly, the two QTLs did not interfere with each other concerning the synthesis of the two substances, pointing to an independent synthesis of the two closely related compounds (Lee et al., 1998). Another flavonoid with activity against *Helicoverpa zea* is isoorientin. In a corn inbred line with high concentrations of isoorientin in the silk it was shown that this was based on the presence of a single recessive gene (Widstrom and Snook, 1998).

In response to nematode invasion, oats (*Avena sativa*) reacts with the induction of flavone-C-glycosides as identified by MS. One of these compounds, O-methyl-apigenin-C-deoxyhexoside-O-hexoside, turned out to be an effective protectant against two major nematodes of cereals, *Pratylenchus* and *Heterodera* (Soriano et al., 2004).

### ALKALOIDS

The best-known alkaloids of grasses are hordenine (*N,N*-dimethyltriamine) and gramine (*N,N-*dimethylindolemethyl-amine), respectively. Hordenine is found in many plant species and in cereals it has been reported in barley, millet (*Panicum miliaceum*) and sorghum (*Sorghum vulgare*; Smith, 1977). Both alkaloids have been shown to act as feeding deterrents against grasshoppers (Hinks and Olfert, 1992). Feeding tests with specialist (*Heliothis subflexa*) and generalist (*Heliothis virescens*) caterpillars also showed deterring effect of hordenine on the feeding behavior and, interestingly, *Heliothis subflexa* was more affected than *Heliothis virescens* (Bernays et al., 2000). Gramine also influences the feeding behavior of aphids. Feeding experiments with *Schizaphis graminum* and *Rhopalosiphum padi *on barley seedlings revealed that the concentration of gramine in the plant and also its tissue location were affecting the feeding behavior (Zúñiga et al., 1988).

These above-mentioned examples were not based on metabolome-covering studies but concentrated specifically on compounds acting as feeding deterrents or with toxic properties. A recent attempt to get a more global picture of herbivore-induced changes in the metabolome of maize was published by Marti et al. (2013). Using UHPLC LC-time-of-flight mass spectrometry (UHPLC-TOF-MS) they took an unbiased approach to determine changes in the metabolite profile at the local and systemic level in maize plants infestated with *Spodoptera littoralis*, thus revealing 32 differentially regulated compounds. It is to be expected that the availability of novel methodologies will speed up our knowledge on the changes occurring at the metabolic level in various plant–insect interactions.

## CURRENT METABOLOMIC TECHNOLOGIES

Few analytical techniques are able to profile a broad range of metabolites in a single analysis. An ideal method that would detect, quantify, and identify all metabolites present in a given plant with high sensitivity, dynamic range, and reproducibility, does not exist (Dunn, 2008; Wolfender et al., 2009). The most comprehensive methods can detect a few thousands of markers, of which only a small portion may be identified (Obata and Fernie, 2012). Amongst the detectors that may be considered for metabolomics, two unarguably stand out from the crowd, namely MS and NMR. In this section, a brief description of both methods is presented with an emphasis on their advantages and limitations and the latest developments in the respective fields.

### MS-BASED METHODS

Mass spectrometry involves the generation of ions and the measurement of their mass-to-charge ratio, providing structural information on the detected molecules. MS may be used either alone or coupled with separation techniques including gas chromatography (GC), LC, and capillary electrophoresis (CE). Contrary to NMR (see below), MS is a highly versatile technique with numerous combinations of ionization sources and analyzers possible. However, no combination is as universal as NMR and therefore the chosen approach may have a strong impact on the classes of metabolites detected. The main advantage of MS over NMR is its extreme sensitivity that allows for detection of metabolites present in trace amounts (Dettmer et al., 2007). Another advantage, in particular when hyphenated to separation techniques, is its capacity to separate compounds in complex mixtures with high resolution. Finally, MS has proved very efficient for the analysis of certain specific classes of metabolites such as lipids and is thus accepted as the method of choice in lipidomics. In contrast, absolute quantification of signals is not possible in the absence of reference standards because ionization is compound-dependent. Finally, in comparison to NMR, the relatively poor reproducibility of MS may render its use in long-term studies problematic when samples cannot be stored for a prolonged period of time (Glauser et al., 2013).

### DIRECT MS

Direct MS represents an interesting approach for high-throughput fingerprinting of large numbers of biological samples. In general, high resolution mass spectrometers are employed because of their important separative power (Dettmer et al., 2007). Three types of analyzers may be employed: TOF, electrostatic trap, or Orbitrap, and Fourier transform ion cyclotron resonance (FT-ICR). Currently, TOF, Orbitrap, and FT-ICR can attain maximal resolving powers of 30’000–60’000, 240’000, and >1’000’000, respectively. Contrary to TOFs, the two latter technologies allow for the resolution of fine isotopic distributions (e.g., ^13^C_2_ and ^34^S isotopes) and are certainly the methods of choice in direct MS metabolomics. However, such resolving powers can only be achieved at low scanning rates, preventing their use in combination with fast chromatographic techniques (Hopfgartner, 2011; Glauser et al., 2012). This is obviously not an issue in direct MS where scan times of 2–5 s may easily be implemented without sacrificing throughput or resolution.

Several ionization methods may be used, including atmospheric pressure ionization (API) methods such as electrospray (ESI), AP chemical ionization (APCI), and AP photo-ionization (APPI) where samples are usually either injected in the so-called flow-injection (FI) mode, or infused at a constant flow rate, a process referred to as direct infusion (DI) mode. Recently, ambient approaches have been developed for the analysis of liquid or solid samples, e.g., desorption-ESI (DESI), desorption-APCI (DAPCI), or extractive-ESI (EESI), and represent promising tools for direct MS metabolomics. Direct analysis in real time (DART) which also operates at AP but relies on different phenomena, also presents interesting features for metabolomics. However, these techniques have only been used in a very limited number of studies (Lee et al., 2012) and more evidence of their applicability to comprehensive plant metabolomics is needed. Another technique complementary to API methods is matrix assisted laser desorption/ionization (MALDI). While MALDI has been traditionally used in proteomics due to its capacity to analyze biomolecules, its use in plant metabolomics has been so far rather limited. The main reasons are the difficulty to produce ions from the relatively hydrophobic species present in plant tissues (Cha et al., 2008), and the fact that the matrices necessary for MALDI generate high background noise in the low mass region of the spectra which may interfere with small metabolites (Shroff et al., 2009). Nevertheless, ion-free matrices (e.g., DIOS, for desorption/ionization on silicon) or rational protocols for matrix selection (Shroff et al., 2009), are potential alternatives for the use of laser induced desorption/ionization in metabolomics. Moreover, as DESI, MALDI can be employed as a “microscope” by collecting mass spectra over a sample surface and reconstructing MS data as an image, a process called MALDI imaging. This method shows great promise for the study of the spatial distribution of metabolites within plant tissues or at the single cell level and is expected to play an increasing role in the future.

### HYPHENATION TO SEPARATIVE METHODS

The coupling of MS to separative methods is a powerful means to improve resolution and marker detection by providing multidimensional data (e.g., 3D data consisting of *m/z* ratios, retention times and peak areas). Isomers may be distinguished, and ion suppression effects much reduced. GC and LC are the two most frequently used chromatographic techniques in MS-based metabolomics. Moreover, another separation method, CE, is gaining interest for the analysis of polar metabolites.

The coupling between GC and MS was achieved long before that of LC and MS and was already used in the early 1970s for human metabolite profiling (Horning et al., 1971) and in the 1980s for plant analysis (Sauter et al., 1991). In the domain of crops, it has been used e.g., to screen wheat cultivars resistant to FHB (Hamzehzarghani et al., 2005). Only volatile and thermally stable molecules can be analyzed by GC-MS. In other words, volatile metabolites such as mono- or sesquiterpenes, small aldehydes, and alcohols may be directly analyzed without chemical modification. However, the vast majority of plant metabolites is not volatile and requires chemical derivatization to increase volatility and thermal stability before GC-MS analysis. This is for instance the case for primary metabolites such as mono- or disaccharides, amino acids, organic acid, and fatty acids. Most often, a two-stage derivatization process is employed: carbonyl groups are first converted to oximes derivatives using e.g., methoxyamine hydrochloride-HCl, followed by formation of trimethylsilyl (TMS) esters with silylating reagents, typically N-Methyl-N-(trimethylsilyl)trifluoroacetamide (MSTFA; Lisec et al., 2006). It has been shown that temperature and derivatization time may affect the outcome of the results, that a range of derivatization products may be formed from a single metabolite, and that the sample stability is a concern (Dunn and Ellis, 2005). Despite these facts, GC-MS after derivatization is nowadays accepted as a gold standard in the field of metabolomics. This is certainly due to the fact that, when coupled through electron ionization, GC-MS yields reproducible and typical spectra, which has enabled the creation of spectral libraries containing hundreds of thousands of mass spectra. By performing mass spectral searches against these libraries, metabolite identification may be successful. However, these libraries are not totally exhaustive and structural identification *via* the interpretation of fragment ions is sometimes necessary. Recent trends in the field of GC comprise the development of 2D GC (GCXGC) metabolomic methods for increased resolution and selectivity (Pierce et al., 2006), and that of fast GC methods using shorter and narrower columns for increased throughput (Jonsson et al., 2004).

In plants, a large portion of metabolites remains inaccessible to GC-MS. For example, flavonoid glycosides or BX glycosides are two important classes of defense secondary metabolites that cannot be volatilized even after derivatization. In such context, the use of LC-MS as an alternative to GC-MS must be considered. With LC-MS, minimal sample preparation is required and the range of metabolites that can be covered is theoretically much wider than that of GC-MS. In principle, LC-MS may detect most organic compounds except extremely volatile ones. For this, several different chromatographic modes shall be employed. Reverse-phase (RP) chromatography using C18 columns has been largely adopted in metabolomic studies. This mode is suitable for most plant secondary metabolites that generally display mildly polar properties (Allwood and Goodacre, 2010). However, very polar and very hydrophobic species require other modes of LC. The former are best analyzed by hydrophilic interaction LC (HILIC; Tolstikov and Fiehn, 2002), while the latter are traditionally separated by normal phase (NP) LC using non-polar solvents such as tert-butyl methylether or hexane. LC and MS are usually interfaced with API sources, predominantly ESI and less often APCI or APPI. These soft ionization techniques yield ions of the molecular species (M+H)^+^ in positive mode, and (M-H)^-^ in negative mode, and various adducts, multimers or multiply charged ions. In APCI and APPI, radical cations (M)^+^ or anions (M)^-^ may also be formed. Recently, sub-2 μm stationary phases and chromatographs able to withstand pressures up to 1300 bars have been introduced on the market. Such systems are referred to as UHPLC and offer a substantial improvement in chromatographic performances, either for the enhanced resolution of complex extracts or the analysis of numerous samples in a short time (5–15 min per sample (Eugster et al., 2011)). The number of publications which report the use of UHPLC-MS for metabolomic studies has grown exponentially over the last years and the trend will definitely not be reversed in the near future. Still, whatever powerful they are, LC-MS and UHPLC-MS cannot replace all other techniques because they also present some limitations, such as the problem of ion suppression and the lack of reproducibility of fragmentation spectra which complicates the creation of mass spectral libraries based on LC-MS data (Glauser et al., 2013).

Capillary electrophoresis mass spectrometry can be viewed as an alternative to HILIC-MS for polar or charged metabolites. The principle of CE involves the separation of molecules according to their mass-to-charge ratio under the influence of an electric field. To date, CE-MS has been relatively rarely employed in plant metabolomics (Sato et al., 2004). Nevertheless, its different selectivity compared to GC and LC makes it a promising tool for the analysis of charged species and further applications may be anticipated in the future (Ramautar et al., 2009).

### NMR-BASED METHODS

Nuclear magnetic resonance is a universal non-destructive and high-throughput technique that requires minimal sample preparation. Generally, plant samples are either freeze-dried and directly extracted in a mixture of D_2_O-CD_3_OD buffered at e.g., pH 6.0 (Kim et al., 2010), or extracted fresh with HClO_4_ 1M with subsequent freeze-drying and redissolution in D_2_O (Kruger et al., 2008). Standard extracts such as those prepared for LC-MS analysis may also simply be evaporated and redissolved in an appropriate deuterated solvent provided that they are concentrated enough. The identification of markers of interest relies on the comparison of specific NMR chemical shifts for plant metabolites with those of reference compounds under identical solvent conditions. A main advantage of NMR over MS is that the signal intensities can be directly linked to the concentration of metabolites, which makes NMR an absolute quantitative method. A majority of applications has used ^1^H-NMR due to the omnipresence of hydrogen atoms in organic molecules, the relatively good sensitivity of NMR for their detection compared to ^13^C or ^15^N, and the speed of analysis. However, ^1^H-NMR spectra are often crowded and the detection of certain metabolites may be hindered or biased due to overlapping signals (Kim et al., 2010). An increase in resolution is therefore desirable and may be achieved by the use of stronger magnets (up to 1 GHz for hydrogen atoms), complementary 2D experiments such as *J*-resolved (requiring longer analysis times), or LC-NMR approaches. Another drawback is the lack of sensitivity (several orders or magnitude lower than that of MS), although the use of cryogenic and/or micro probes may increase sensitivity by a factor of 20 (Kim et al., 2010). Still, NMR is superior to MS in terms of reproducibility (Verpoorte et al., 2007; Schripsema, 2010), which makes it an interesting tool for the measurement of predominant constituents of plants such as sugars, amino acids, organic acids, and major secondary metabolites (Wolfender et al., 2013). Recently, an interesting study reported the comparison of GC-MS and NMR performances for metabolite profiling of rice samples (Barding et al., 2012). While GC-MS proved as expected much more sensitive and could detect several minor primary metabolites not observed by NMR, it also presented some limitations including low dynamic range and failure to detect certain metabolites such as dipeptides. Finally, NMR analysis may also be used to complement UHPLC-MS to assess the functional groups and the final identity of purified compounds, such as the BX derivatives in maize (Ahmad et al., 2011).

### DATA PROCESSING AND MINING

All “omics” approaches heavily rely on bioinformatic tools for the analysis of the large datasets generated and metabolomics is not an exception. In the case of GC-MS or LC-MS datasets for example, raw data must be recorded and converted to appropriate formats for further data handling, including noise filtering, peak detection, and alignment. Such processing procedure aims to obtain homogenous information for a straightforward comparison of multiple samples by statistical methods. Results are displayed in the form of a marker table containing sample names, variables (characterized by *m/z* and retention time values) and peak intensities or areas. Each sample should ideally be defined by the same number of variables and each variable should correspond to the same metabolite. This peak picking procedure may be achieved using a range of free packages, e.g., MarVis^1^, MzMine (Katajamaa and Oresic, 2005), XCMS (Smith et al., 2006), MetAlign (Lommen, 2009), or commercial softwares, e.g., Markerlynx^2^.

In a second step, multivariate analysis methods may be used to reduce the dimensionality of data, revealing clusters of samples, and discriminatory variables. Prior to this, a pre-treatment of the data is often carried out to provide suitable data for further analysis. Normalization to the total integrated area or to a given internal standard may or may not be applied to the dataset depending on the biological model studied. Scaling enables the adjustment of the weight of each variable in the model (e.g., unit variance or Pareto scaling). Principal component analysis (PCA) is a common unsupervised multivariate method used for exploratory analyses by building principal components describing the maximal variance of data (Hotelling, 1933). PCA has been employed in the majority of metabolomic studies and represents a good starting point for exploring metabolomic data. Projection to latent structures by means of partial least squares (PLS; Wold et al., 2001) is a well-known supervised regression method and is often employed to maximize the separation between classes. Several other statistical methods exist and interested readers are invited to consult specialized literature for further information (e.g., Boccard et al., 2010; Liland, 2011).

## CONCLUSION AND OUTLOOK

The work summarized in this review illustrates the pivotal role of metabolites in cereals during various biotic stresses. Within plant-biotroph interactions such as *Magnaporthe grisea* infection on rice, amino acids as well as sugars are known to be induced (Jones et al., 2011). A more extensive knowledge is also available for maize–pathogen interactions. Analysis of tissue-specific infections of maize with *Ustilago maydis* uncovered a prominent induction of the shikimate and flavonoid pathways in response to fungal attack (Doehlemann et al., 2008). Recently, the organ-specific metabolome changes of maize during infections with the hemibiotrophic fungus *C. graminicola* have been described (Balmer et al., 2013), uncovering higher levels of defense-associated metabolites including flavonoids in roots compared to leaves. In response to FHB, resistant barley lines were found to employ much higher levels of metabolites belonging to the flavonoid, phenylpropanoid, fatty acid, and terpenoid pathways compared to susceptible lines (Choo, 2006). Interestingly, recent evidence was also presented that BX, in addition to their toxic effects, function as a signal in maize immunity (Ahmad et al., 2011).

Recent advances in transcriptomic and metabolomic technologies facilitate a novel trend of integrated “omics,” where cereals are screened in regard to pathogen-resistant genotypes as well as biochemical phenotypes (Langridge and Fleury, 2011). A combined transcriptomics/metabolomics analysis of maize and barley infected with different pathogens showed that the transcriptional reprogramming upon pathogen attack does not necessarily correlate with adaptation of the primary metabolism (Voll et al., 2011). Moreover, metabolomic profiling techniques are also applicable for evaluating genetically modified cereals (Ricroch et al., 2011). For instance, a transgenic barley line expressing a chitinase was compared to non-transgenic lines (Kogel et al., 2010). In a recent study of genetically modified maize, Barros et al. (2010) compared the transcriptome, proteome, and metabolome of different lines exposed to variable environmental factors. In this particular example, these factors affected the metabolome much stronger than genetic modification. Nevertheless, metabolomics is a useful tool for screening crops for pathogen resistance, as shown in the case of barley lines screened for resistance against *Gibberella zeae* (Kumaraswamy et al., 2011). There, 161 metabolites could be associated with less susceptible barley lines, including linoleic acid, p-coumaric acid, and naringenin. Besides its utility to screen for resistance traits, metabolomics is also widely applied as diagnostic tool. For instance, metabolomic analysis of naturally contaminated oat, rye and barley grains yielded distinct patterns of metabolites in infected versus non-infected grains (Perkowski et al., 2012). Moreover, in the same study, mycotoxins could also be analyzed in parallel to the plant metabolites, demonstrating the power of metabolomics as diagnostic aid.

Considering the great potential of cereal metabolomics in pathogen and pest resistance, it is not surprising that targeting metabolomic pathways is part of recent transgenic strategies in different cereals, mainly in rice. For instance, a series of momilactone A over-accumulating lines were generated (Sawada et al., 2004; Mori et al., 2007; Kurusu et al., 2010). Some of these lines exhibit enhanced resistance against *Magnaporthe grisea* and *Xanthomonas oryzae*. Similarly, overexpression of sakuranetin in rice resulted in an increased resistance to *Magnaporthe grisea* (Kim et al., 2009). Thus, manipulating biosynthetic pathways of metabolites appears to be an opportunistic strategy in transgenic crop enhancing programs. However, this approach is considered to also have a major drawback, namely the possible manipulation of metabolomic fluxes (Hassan and Mathesius, 2012). For instance, manipulating the phenylpropanoid metabolism in *Medicago truncatula* affected lignin synthesis in roots (Laffont et al., 2010). Moreover, an imbalance of secondary metabolites could possibly result in negative effects for the plant, including disadvantageous transport or exudation defects, as well as negative physiological costs (Hassan and Mathesius, 2012).

Metabolomics research is also accompanied by major limitations, the most important one being the current inability to analyze the entire metabolome. The number of plant metabolites is estimated to 200’000 or more (Trethewey, 2004; Saito and Matsuda, 2010) and the identified compounds summarized in public databases represent only a very little sample of this great variety. Thus, most of the compounds detected in current metabolomics studies remain unidentified. Despite the existence of public MS-databases such as KNApSAcK^3^, KEGG^4^, or BRENDA^5^, updating and combining the information is one of the major future challenges. An additional drawback is the limited range of metabolites that can be analyzed simultaneously. For instance, excessive levels of sugars can interfere with the detection of flavonoids (Sumner et al., 2003). Moreover, metabolite profiling techniques usually need to be adapted according to the compounds of interest; for example, oligosaccharides are difficult to analyze using LC/MS (Sumner et al., 2003). Finally, it has to be considered that diseased plant material poses a special challenge to the methodology that can be applied and might require specific approaches (Allwood et al., 2012). As a consequence, analytical approaches need to be optimized for a given experimental setup. Another main challenge of metabolomics is the bioinformatics aspect, including data analysis and storage. As a high-throughput technology, current metabolomics generates massive amounts of datasets. The examination of such sets requires appropriate statistical models, as well as appropriate data visualization approaches (Sumner et al., 2007). In addition, the challenge is also to filter biological meaning out of massive datasets, especially when looking at entire metabolomes rather than selected markers. Further advances in bioinformatic tools combining general “omics” will contribute to a better understanding of the role of cereal metabolites during biotic stresses. This knowledge is expected to have a great impact in designing future cereal crop enhancement projects.

## Conflict of Interest Statement

The authors declare that the research was conducted in the absence of any commercial or financial relationships that could be construed as a potential conflict of interest.

## Footnotes

1 http://marvis.gobics.de

2 http://www.waters.com

3 www.kanaya.naist.jp/KNApSAcK/

4 www.genome.ad.jp/kegg/kegg2.html

5 www.brenda.uni-koeln.de
